# Prevention of malaria in pregnancy: The threat of sulfadoxine-pyrimethamine resistance

**DOI:** 10.3389/fped.2022.966402

**Published:** 2022-08-18

**Authors:** Sesh A. Sundararaman, Audrey R. Odom John

**Affiliations:** Department of Pediatrics, Children's Hospital of Philadelphia, The Perelman School of Medicine at the University of Pennsylvania, Philadelphia, PA, United States

**Keywords:** malaria, drug resistance, low birth weight, antimalarial, IPTp

## Abstract

Malaria infection in pregnancy can lead to adverse outcomes for both the pregnant person and fetus. The administration of intermittent preventative therapy (IPTp) with sulfadoxine-pyrimethamine (SP) during pregnancy (IPTp-SP) improves outcomes, including severe maternal anemia, placental malaria infection, and low infant birth weight. The WHO recommends IPTp-SP for pregnant individuals living in areas of moderate or high malaria transmission in Africa. The current regimen consists of two or more doses of SP starting as early as possible in the second trimester, at least 1 month apart. Unfortunately, rising *Plasmodium falciparum* SP resistance throughout Africa threatens to erode the benefits of SP. Recent studies have shown a decrease in IPTp-SP efficacy in areas with high SP resistance. Thus, there is an urgent need to identify new drug regimens that can be used for intermittent preventative therapy in pregnancy. In this review, we discuss recent data on *P. falciparum* SP resistance in Africa, the effect of resistance on IPTp-SP, and studies of alternative IPTp regimens. Finally, we present a framework for the ideal pharmacokinetic and pharmacodynamic properties for future IPTp regimens.

## Introduction

Malaria, caused by parasites of the genus *Plasmodium*, is a major cause of morbidity and mortality across the globe (1). The majority of malaria-related deaths occur in sub-Saharan Africa, and are caused by one parasite species, *Plasmodium falciparum*. Children, especially those under the age of five, are at highest risk of severe disease. Partial immunity, which develops through repeated exposure, provides some protection against both symptomatic and severe disease (2).

While the risks of malaria decrease with age and regular reinfections, they recur again in pregnancy, where both symptomatic and asymptomatic infection have significant consequences for the pregnant person and fetus. This burden is felt disproportionally by pregnant people in sub-Saharan Africa, where over 10 million pregnant individuals are likely exposed to malaria each year (1). Intermittent presumptive therapy for malaria in pregnancy (IPTp) with sulfadoxine pyrimethamine (SP) decreases the adverse effects of malaria in pregnancy, but the benefits of this intervention are threatened by increasing SP resistance throughout sub-Saharan Africa (3). New antimalarial therapies are, therefore, needed to ensure continued protection of pregnant people and fetuses.

## Malaria in pregnancy and intermittent preventative therapy

The WHO estimates that over 10 million pregnant individuals (34% of all pregnancies worldwide) are exposed to malaria each year (1). The risks of malaria are increased in pregnancy (4, 5). Not only does pregnancy represent a state of transient immunosuppression, the malaria parasite *Plasmodium falciparum*, expresses a unique adhesion factor, var2csa, that binds chondroitin sulfate on the placenta (6). Parasite adhesion to the maternal surface of the placenta leads to increased inflammation and reduced placental blood flow (6). For these reasons, placental malaria contributes to poor outcomes for both the birthing parent and developing fetus. Malaria during pregnancy contributes to maternal anemia, low birthweight, intrauterine growth restriction, preterm delivery, stillbirth, and death in the neonatal period (7–9). In spite of these many potential complications, pregnant individuals may yet present with few or no symptoms, making prompt identification and treatment of infection difficult (5). Early studies showed that antimalarial prophylaxis in pregnancy reduced maternal anemia and increased infant birthweight (10, 11). This led the WHO to recommend that pregnant people in malaria endemic regions receive intermittent antimalarial prophylaxis.

Currently, intermittent preventative therapy for malaria in pregnancy (IPTp) consists of treatment doses of sulfadoxine pyrimethamine (SP), starting in the second trimester (12). IPTp-SP improves outcomes for both the pregnant person and fetus. Early clinical trials of SP during pregnancy showed a marked reduction in peripheral parasitemia, maternal anemia, placental malaria, preterm birth, and an increase in infant birth weight (13–16). Additional studies showed that three or more doses of SP, given at least four weeks apart, further decreases the overall prevalence of low birth weight and preterm birth (17, 18). The WHO estimates that current levels of IPTp coverage prevent over 400,000 cases of low birthweight each year.

## Resistance to sulfadoxine pyrimethamine

**Figure 1:**
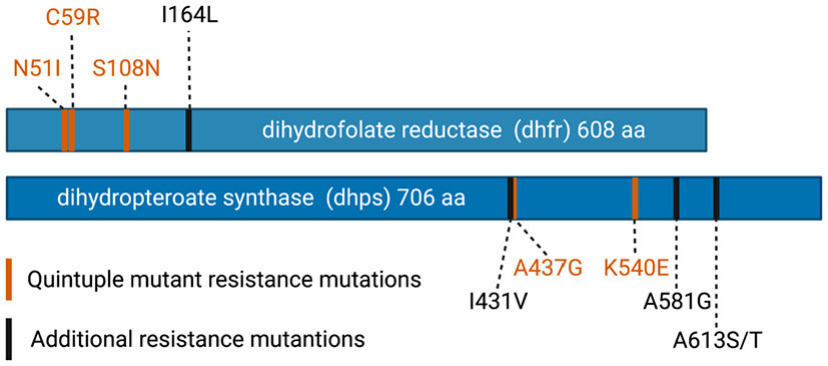
Mutations in dihydrofolate reductase (dhfr) and dihydropteroate synthase (dhps) that confer resistance to sulfadoxine pyrimethamine. Mutations found in the highly resistant “quintuple mutant” are shown in orange. Additional mutations, that are increasing in prevalence in Africa, are shown in black. Figures generated in Biorender.

The benefits of IPTp-SP in pregnancy are threatened by rising SP resistance in *P. falciparum*. Sulfadoxine and pyrimethamine both inhibit folate synthesis in malaria parasites, acting on dihydropteroate synthase (*dhps*) and dihydrofolate reductase (*dhfr*), respectively. SP resistance develops through the accumulation of mutations in these enzymes (Figure 1). In East Africa, parasites containing a combination of three distinct *dhfr* mutations (N51I, N59R, and S108N) and two *dhps* mutations (A437G, and K540E) have become highly prevalent. The presence of these parasites, termed “quintuple mutants,” is highly predictive of treatment failure of clinical malaria in sub-Saharan Africa (19, 20). Acquired immunity likely also plays a role, as treatment failure is observed in young children infected with less resistant parasites (21).

While the presence of quintuple mutants predicts poor treatment response in cases of acute uncomplicated malaria, *P. falciparum* continues to evolve higher levels of resistance. In East Africa, additional mutations, including *dhps* A581G, *dhfr* I164L, and *dhps* A613S/T have begun to emerge. These mutations, when present on the quintuple mutant background, further increase SP resistance and increase the risk of clinical treatment failure (3, 22). A581G and A613S/T mutations have also been detected in West Africa, in the absence of K540E (23). While the lack of K540E in these parasites increases their susceptibility to SP, the risk of highly resistant parasites emerging in West Africa, through the acquisition of K540E or other novel mutations, remains.

## The effects of sulfadoxine pyrimethamine resistance on IPTp-SP

Early studies of IPTp-SP in areas of increasing SP resistance showed continued benefit. A 2007 meta-analysis by Kuile, et. al. found that IPTp-SP maintained effectiveness in preventing low birth weight, maternal anemia, maternal parasitemia at delivery, and placental malaria, even in geographic regions where SP treatment failure rates in children with acute malaria ranged from 9–39% (24). Importantly, treatment failure in children appears to occur at lower levels of SP resistance than in adults. Thus, pediatric malaria treatment failure rates are a surprisingly poor proxy for the effectiveness of ITPp-SP in pregnancy (21). More recent studies, using genetic markers of SP resistance, have found that IPTp-SP effectiveness is indeed reduced in areas where highly resistant parasites are prevalent. Van Eijk, et. al. found that the relative risk reduction of IPTp-SP on malaria-associated pregnancy outcomes decreased with increasing prevalence of the K540E mutation (25). While IPTp-SP confers some benefit in areas where the prevalence of K540E was >90%, the benefit was lost in areas where A581G prevalence was >10%. The loss of effectiveness, due to A581G, was also observed by Chico et al. (26). Given the increasing prevalence of A581G in East Africa (23), these data suggest that decreased IPTp-SP effectiveness will soon be widespread in this region.

## Dihydroartemisinin-piperaquine as an alternative to IPTp-SP

As the efficacy of IPTp-SP wanes due to increasing resistance, there is an urgent need to identify alternative pharmaceutical strategies for preventing adverse pregnancy outcomes from malaria. While clinical studies have examined multiple alternatives, including amodiaquine, mefloquine, azithromycin, and chloroquine, most have failed due to adverse effects or lack of benefit (27–29). Recently, combination therapy with dihydroartemisinin plus piperaquine (DP) has been proposed as a replacement to IPTp-SP, given the tolerability and rapid, potent activity against asexual *P. falciparum*. IPTp-DP reduces the prevalence of both clinical and sub-patent malaria infection as compared to IPTp-SP (30–32). This has not, however, translated to a consistent improvement in birth outcomes. Thus, far, three randomized controlled trials have found IPTp-DP to be equivalent to IPTp-SP, and only one has found it to be superior in preventing adverse birth outcomes (30–33).

The lack of benefit of IPTp-DP, relative to IPTp-SP, for birth outcomes, in spite of a decrease in detectable peripheral parasitemia, suggests that the latter may not adequately reflect pathology at the placenta (33, 34). Indeed, randomized controlled trials comparing IPTp-SP to intermittent screening and treatment (IST), where pregnant individuals are screened by rapid diagnostic test or peripheral smear and treated only if positive, have found IST to be inferior (33, 35). Moreover, an analysis of over 1,500 patients found that placental malaria infection was associated with lower birth weight regardless of whether parasites were detected in the peripheral blood, and that the presence of peripheral parasitemia, without placental infection, was not associated with lower birth weight (36).

There are multiple possible explanations for the lack of consistent benefit of IPTp-DP over IPTp-SP. First, levels of SP resistance may not have reached thresholds that compromise IPTp-SP. To date, trials comparing IPTp-DP to IPTp-SP have been conducted in areas where the prevalence of A581G and other highly resistance genotypes is low (0–5.8%) (31, 33, 37, 38). In the absence of these highly SP resistant parasites, it is possible that IPTp-SP and IPTp-DP are equally effective in preventing adverse birth outcomes. Second, the frequency of DP dosing may be insufficient to maintain protection. Pharmacokinetic analyses of pregnant individuals given IPTp-DP found that higher exposure to piperaquine was associated with reduced odds of placental malaria, preterm birth, and low birth weight (39). Future studies of IPTp-DP may need to test alternative DP dosing regimens, such as weekly administration, and should be focused in areas of high SP resistance (39).

## Future directions

The decreasing effectiveness of SP in East Africa, and lack of a clear alternative IPTp regimen, highlight the importance of continued research into IPTp options. Important areas of focus will include the development of novel therapeutics, establishment of drug resistance markers that correlate with loss of IPTp effectiveness, and discovery of non-invasive methods to detect the presence of placental malaria infection prior to delivery.

### Development of novel therapeutics

**Figure 2:**
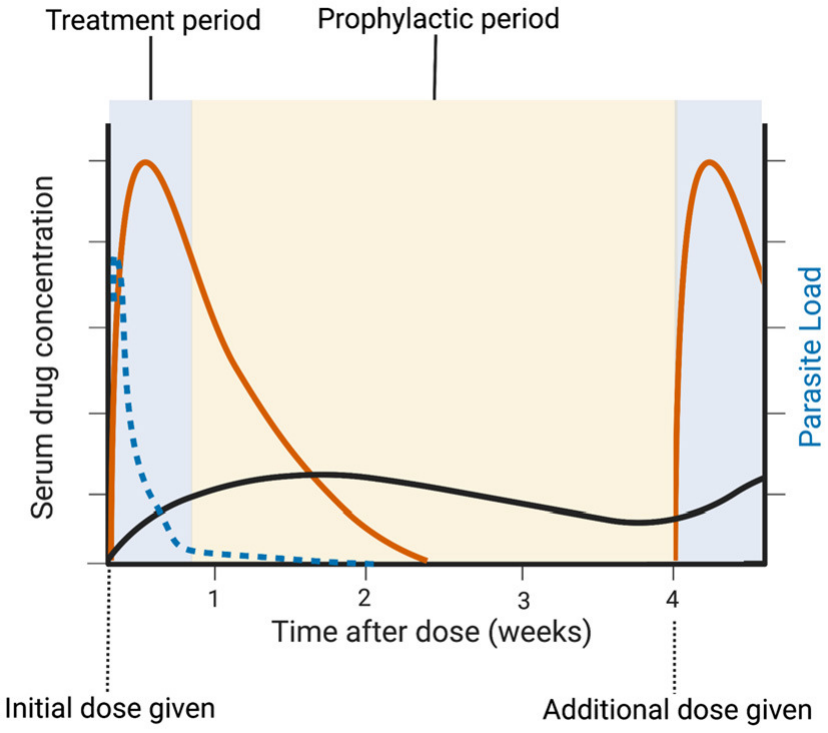
Idealized IPTp regimen. A treatment dose of a drug that meets MMV TPP-1 (management, orange line) eradicates existing asexual and placental infection (dotted blue line). A drug that meets TPP-2 (chemoprevention, black line) provides long-term protection against re-infection until the next prenatal visit.

If DP proves superior to SP for IPTp, its usefulness may unfortunately be short-lived due to evolving resistance patterns in *P. falciparum*. There is evidence for emerging artemisinin resistance in Africa, and piperaquine resistance in South East Asia (40, 41). The rise of both SP and DP resistance highlights the need for continued development of new antimalarials for the treatment of clinical malaria and intermittent preventative treatment in pregnancy. To address this ongoing need, the Medicines for Malaria Venture (MMV) has proposed two Target Product Profiles (TPPs) for antimalarial drug development (42). TPP-1 applies to medications for acute malaria treatment, with essential parameters that include activity against resistant parasites, rapid onset of action, and a large (>12 log_10_) reduction in asexual parasite load. TPP-2 applies to medications for chemoprotection, with essential parameters that include a long dosing interval (weekly or longer) and efficacy against the pre-erythrocytic liver stages. The clinical benefit of IPTp-SP is likely due to both eradication of any ongoing malaria infection and temporary prophylaxis against new infection (43); both sulfadoxine and pyrimethamine remain detectable in serum for more 40 days after dosing (44). An ideal IPTp regimens should thus aim to meet both TPPs for optimum benefit (Figure 2). However, antigametocyte and antihypnozoite activities, to eradicate the sexual transmission stages or the latent liver stages of *P. vivax* or, respectively, are not necessary for IPTp.

### Drug resistance markers to predict IPTp effectiveness

While combined *dhps* K540E and A581G mutations are an important genetic marker of decreasing IPTp-SP effectiveness in East Africa, the generalizability of these markers to other parts of Africa will likely be limited. There is significant variability in the prevalence of *dhfr* and *dhps* resistance mutations across the continent. In West Africa, the A581G mutation is found in the absence of K540E and is associated with increased susceptibility to SP, relative to parasites containing both mutations (3, 25, 45, 46). However, other mutations, such as *dhps* I431V, appear to be emerging. Additional studies are necessary to determine the effects of this I431V on the effectiveness of IPTp-SP. Phenotypic drug resistance studies of field isolates to SP, will also be useful. While traditional *in vitro* assays are limited by the substantial technical challenges of culture-adapting large numbers of field isolates, short-term *ex vivo* drug sensitivity assays can be used to phenotypically screen fresh clinical isolates (47–49). The identification of *ex vivo* phenotypic markers, such as MIC or IC_50_, that correlate with clinical IPTp-SP failure could allow for generalizability to areas with distinct *dhfr* and *dhps* genetic backgrounds.

### Detection of placental malaria infection

Current studies of IPTp are hindered by the inability to assess the presence and degree of placental malaria infection. While the goal of IPTp is to improve outcomes for both the pregnant person and fetus, confounders make it difficult to monitor the clinical effectiveness of IPTp and to compare it across populations or studies. Accurate identification of placental malaria requires labor-intensive histopathology or studies of placental blood, and can only be performed after delivery. Data from studies of IPTp-DP suggest that peripheral parasitemia, even when identified by molecular methods, may overestimate effects on placental infection (33, 36). Future studies would benefit from the identification of new biomarkers of placental infection. These could be derived either from infecting parasites (i.e., levels of var2csa antigen), or the host (i.e., profiles of inflammatory cytokines) (50). Such biomarkers would facilitate longitudinal monitoring of the effectiveness of current IPTp regimens, identification of phenotypic or genotypic resistance markers that predict IPTp treatment failure, and comparisons of new IPTp regimens to current standard of care. They would also help differentiate the benefits conferred by IPTp from other pregnancy interventions, improving our understanding of the effects of IPTp in the evolving field of maternal-fetal health.

## Author contributions

AO and SS conceived of this work, and both contributed to drafting and editing the manuscript. All authors contributed to the article and approved the submitted version.

## Funding

AO was supported by NIH/NIAID R01AI103280, R21AI154370, and R21AI144472. AO is an Investigator in the Pathogenesis of Infectious Diseases of the Burroughs Wellcome Fund. Funders had no role in conception or preparation of this manuscript. Institutional funds will be used for open access publication. SS is supported by the PIDS-St. Jude Children's Research Hospital Fellowship Award in Basic and Translational Science.

## Conflict of interest

The authors declare that the research was conducted in the absence of any commercial or financial relationships that could be construed as a potential conflict of interest.

## Publisher's note

All claims expressed in this article are solely those of the authors and do not necessarily represent those of their affiliated organizations, or those of the publisher, the editors and the reviewers. Any product that may be evaluated in this article, or claim that may be made by its manufacturer, is not guaranteed or endorsed by the publisher.
